# Abnormalities of Mitochondrial Dynamics in Neurodegenerative Diseases

**DOI:** 10.3390/antiox6020025

**Published:** 2017-04-05

**Authors:** Ju Gao, Luwen Wang, Jingyi Liu, Fei Xie, Bo Su, Xinglong Wang

**Affiliations:** 1 Departments of Pathology, Case Western Reserve University, Cleveland, OH 44106, USA; jxg712@case.edu (J.G.); lxw404@case.edu (L.W.); jxl1599@case.edu (J.L.); fxx53@case.edu (F.X.); 2 Department of Neurobiology, Shandong University, Jinan 250012, China; bxs103@sdu.edu.cn

**Keywords:** mitochondrial dynamics, neurodegenerative diseases, Alzheimer’s disease, Parkinson’s disease, amyotrophic lateral sclerosis, Huntington’s disease, mitochondrial fusion and fission, mitochondrial trafficking, mitochondrial dysfunction, neurodegeneration

## Abstract

Neurodegenerative diseases are incurable and devastating neurological disorders characterized by the progressive loss of the structure and function of neurons in the central nervous system or peripheral nervous system. Mitochondria, organelles found in most eukaryotic cells, are essential for neuronal survival and are involved in a number of neuronal functions. Mitochondrial dysfunction has long been demonstrated as a common prominent early pathological feature of a variety of common neurodegenerative diseases, including Alzheimer’s disease (AD), Parkinson’s disease (PD), amyotrophic lateral sclerosis (ALS), and Huntington’s disease (HD). Mitochondria are highly dynamic organelles that undergo continuous fusion, fission, and transport, the processes of which not only control mitochondrial morphology and number but also regulate mitochondrial function and location. The importance of mitochondrial dynamics in the pathogenesis of neurodegenerative diseases has been increasingly unraveled after the identiﬁcation of several key fusion and fission regulators such as Drp1, OPA1, and mitofusins. In this review, after a brief discussion of molecular mechanisms regulating mitochondrial fusion, fission, distribution, and trafficking, as well as the important role of mitochondrial dynamics for neuronal function, we review previous and the most recent studies about mitochondrial dynamic abnormalities observed in various major neurodegenerative diseases and discuss the possibility of targeting mitochondrial dynamics as a likely novel therapeutic strategy for neurodegenerative diseases.

## 1. Introduction

Mitochondria are organelles that can be found in most eukaryotic cells and are required for a wide range of cellular processes such as the generation of cellular adenosine triphosphate (ATP), the synthesis of key metabolites, the production of endogenous reactive oxygen species, Ca^2+^ hemostasis, and programmed and unprogrammed cell death [1,2,3]. The brain, at only 2% of the body weight, consumes about 20% of the body’s energy [4]. Due to their limited glycolytic capacity and extremely metabolically active nature, neurons in the brain are energetically demanding cells requiring the delicate maintenance of mitochondrial function [5]. In addition, as highly polarized cells with complex cellular extensions (processes), i.e., dendrites and axons, neurons also need the timely and appropriate transport and distribution of mitochondria to serve as energy power plants and an internal Ca^2+^ storage pool for localized neuronal activities such as synaptic transmission, axonal and dendritic transport, and synaptic vesicle recycling [6,7].

It is conceivable that the disturbance of mitochondrial function can have severe consequences for neuronal function and structure. A large number of studies suggest that reduced brain metabolism or mitochondrial dysfunction are some of the best documented abnormalities and prominent early features in brains of all major neurodegenerative diseases [8]. Notably, metabolic derangements alone are sufficient to cause neurological deficits [9]. As cellular metabolism and mitochondria are closely related, these findings suggest that mitochondrial dysfunction likely plays a central role in the pathogenesis of neurodegenerative diseases. Mitochondria are highly dynamic organelles that undergo continual fusion and fission events, which not only maintain their integrity and quantity, but also serve crucial mitochondrial functions such as ATP production [10], Ca^2+^ homeostasis [11,12], cell death [13,14,15], and reactive oxygen species (ROS) production [16]. Recent findings of widespread mitochondrial fragmentation, along with altered distribution in cell bodies and neuronal processes in common neurodegenerative diseases [17,18], suggest that abnormal mitochondrial fusion, fission, and trafficking dynamics may contribute to mitochondrial dysfunction and neurodegeneration in these devastating diseases. In this review, we will focus on the role of mitochondria dynamic abnormalities in a number of common neurodegenerative diseases.

## 2. Mitochondrial Dynamics: Fission, Fusion and Trafficking

**Figure 1:**
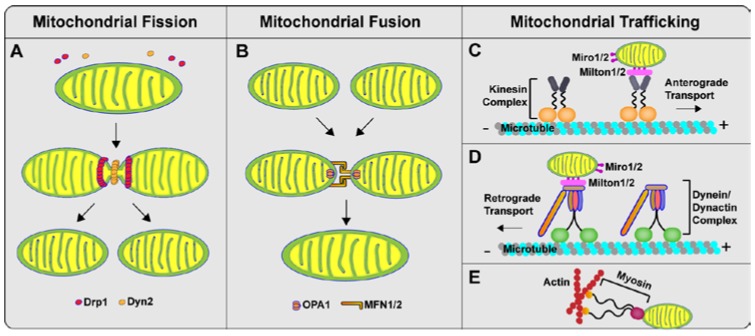
Schematic depiction of mitochondrial dynamics in mammalian cells. (**A**) Cytosolic Drp1 is recruited to the mitochondrial outer membrane by several receptor proteins, followed by oligomerization into ring-like structures to partially constrict the mitochondrial membrane. Then, another dynamin-like protein, dynamin 2 (Dyn2), binds and constricts the mitochondrial membrane further to enable lipid fusion and organelle division; (**B**) The mitochondrial fusion process requires two steps, outer membrane fusion and inner membrane fusion. Outer membrane fusion is mediated through interactions of coiled-coil domains of Mfn1 and Mfn2 to form either homo-oligomeric or hetero-oligomeric complexes to tether membranes together. OPA1 is involved in the formation of cristae junctions as well as in inner membrane fusion; (**C**,**D**) The anterograde motor kinesin-1 and the retrograde motor dynein/dynactin complex directly interact with Milton and Miro on mitochondria to drive their movement along the microtubules; (**E**) Actin motors are associated with mitochondria to facilitate the short-distant movement along the filament.

The morphology of the mitochondrial network is influenced by the delicate balance between opposing fusion and fission events, which are regulated by several large dynamin-related GTPase proteins. The key regulator in the mitochondria fission process is dynamin-related protein1 (Drp1 or DLP1), a large GTPase mainly localized in the cytosol [19]. During fission, cytosolic Drp1 is recruited to the mitochondrial outer membrane by several receptor proteins such as Mff, Fis1, and MiD48/51, followed by oligomerization into a ring-like structure to sever the mitochondrial membrane by self-assembly and GTP hydrolysis [20,21,22,23]. In addition to Drp1, Dyn2, another dynamin-like protein, has been also reported to regulate the final step of membrane division after Drp1 recruitment and polymerization [24] (Figure 1A). The molecular mechanisms responsible for the initiation of mitochondrial fission remain largely unknown. Most recent studies imply that endoplasmic reticulum (ER), together with actin filaments, plays a critical role in the establishment of constriction sites before mitochondrial Drp1 recruitment [25,26]. After the fission process is completed, Drp1 complex remains on one of the daughter mitochondrion [27]. Recent studies indicated that the Drp1 oligomeric complex on mitochondria could not sever mitochondria and become inactive or even inhibitory [28,29,30]. Although the knowledge about the fate of Drp1 oligomeric complex on mitochondria is still limited, our most recent study has reported that the key component of retromer recognition complex VPS35 can preferentially interact with Drp1 oligomeric complex and direct their trafficking from mitochondria to lysosome for degradation [31], implying that the likely recycling of inactive Drp1 oligomeric complex through retromer-mediated endosomal pathway is worthy of further investigation.

Mitochondrial fusion involves the fusion of both the outer and inner membrane and is regulated by at least three other large GTPase proteins, i.e., Mitofusin 1 (Mfn1) and Mitofusin 2 (Mfn2) for mitochondrial outer membrane fusion and optic atrophy protein 1 (OPA1) for mitochondrial inner membrane fusion [32]. Like Drp1, Mfn1, Mfn2, and OPA1 controlled mitochondrial fusion also depends on their self-assembly and GTPase activity. It has been proposed that mitochondrial outer membrane fusion is mediated through interactions of the coiled-coil domains of Mfn1 and Mfn2 to form either homo-oligomeric or hetero-oligomeric complexes to tether membranes together [33,34] (Figure 1B). The outer and inner mitochondrial membrane contains different phospholipids, and the proper phospholipid composition is important for the regulation of mitochondrial fusion [35]. For example, MitoPLD, a divergent family member of the phospholipase D (PLD) family, may regulate mitochondrial fusion by promoting trans-mitochondrial membrane adherence by hydrolyzing cardiolipin, a unique mitochondrial phospholipid predominantly localized in the inner mitochondrial membrane, to generate phosphatidic acid [36]. One recent study showed that mitoguardin (MIGA) proteins can indirectly regulate mitochondrial fusion by facilitating MitoPLD dimer formation [37]. Along this line, cardiolipin has also been reported to control mitochondrial fission by mediating the mitochondrial recruitment and GTPase activity of the key mitochondrial fission factor [38,39]. Nevertheless, determining the molecular mechanism of mitochondrial fusion has proven difficult due to the involvement of both the outer and inner membrane and the likely very complicated processes involved.

In response to various physiological and pathological states, mitochondria are transported to sites with bioenergetics requirements. The positioning of mitochondria at specific cellular locations is regulated mainly by bidirectional (anterograde and retrograde) movements along microtubules for fast movement and along actin filaments for slow movement via different motor-adaptor complexes [40]. The core of the motor-adaptor complex consists of kinesin-1 (also referred to as kinesin heavy chain or Kif5), dynein (cytoskeletal motor proteins), Miro1 and 2 (also known as RhoT1 and RhoT2), and Milton1 and 2 (also known as TRAK1 and TRAK2) [41]. The anterograde motor kinesin-1 and the retrograde motor dynein/dynactin complex directly interact with Milton and Miro on mitochondria to drive their movement along the microtubules [42,43]. In addition to microtubule-based motility, actin filaments are also involved in mitochondria movement [44] (Figure 1C,D). Actin motors, such as Myo2 and Myo19, are associated with mitochondria to facilitate the short-distant movement along the filaments [45,46]. In addition to trafficking, mitochondrial distribution and positioning may be influenced by mitochondrial morphological dynamics [47,48] (Figure 1E). For instance, mitochondrial fission and fusion regulators such as Drp1 and Mfn2 have been reported to regulate mitochondrial axonal transport [49,50,51], therefore suggesting the close interplay between mitochondrial morphological dynamics and trafficking. It is also worth noting that, beyond trafficking within cells, mitochondria can be transported between different cell types. Neurons can release damaged mitochondria and transfer them to astrocytes for degradation [52,53]. Likewise, by a calcium-dependent mechanism involving CD38 and cyclic ADP ribose signaling, astrocytes can release functional mitochondria to be taken up by neurons [54].

## 3. Mitochondrial Dynamics, Mitochondrial Function and Neuronal Function

In addition to their direct effects on mitochondrial shape, length, number, and location, mitochondrial dynamics are critical for the maintenance of the integrity and homogeneity of mitochondria. Impaired mitochondrial fusion has been reported to cause mtDNA point mutations and deletions, which result in the accumulation of dysfunctional mitochondria. It is conceivable that mitochondrial fusion is an important mechanism, allowing the exchange of lipid membrane and intramitochondrial contents between different mitochondria to enable mtDNA repair and equally distribute metabolites to maintain a healthy population of mitochondria [55,56]. Due to the high levels of ROS produced and the lack of efficient DNA repair systems, mitochondria are relatively vulnerable to deleterious damage. The irreversibly defective mitochondria need to be cleared in a timely manner, and mitochondrial fission has been shown to participate in the elimination of damaged mitochondria by autophagy [57]. The mitochondrial genome encodes 13 mRNAs of key subunits of oxidative phosphorylation (OXPHOS) complex I, III, IV, and V [58]. By facilitating mtDNA exchange to complement mutated mitochondrial genes, mitochondrial dynamics are also crucial for mitochondrial bioenergetics. In fact, during transitions between different respiratory states, mitochondrial morphological changes coincide with OXPHOS complex assembly, cristae remodeling, and matrix space condensation [59,60,61,62]. Elongated mitochondrial morphology is usually associated with OXPHOS activity [63]. It is noteworthy that mitochondrial fusion and fission proteins could be directly involved in the assembly of respiratory complexes [64,65], underscoring the important role of mitochondrial dynamics in regulating mitochondrial function.

**Figure 2:**
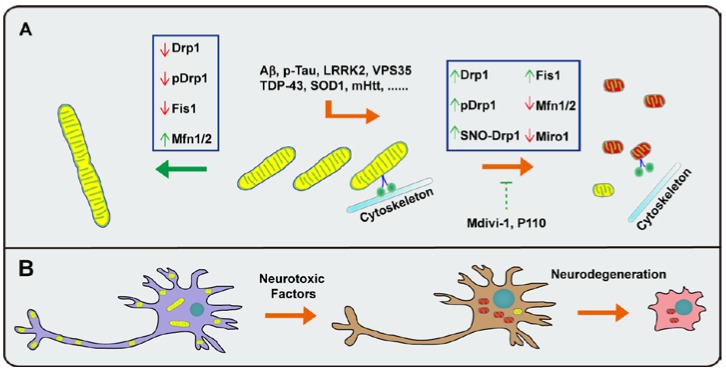
Impaired mitochondrial dynamics in neurodegenerative diseases. (**A**) Mitochondrial fragmentation is a common factor in neurodegeneration, leading to impaired mitochondrial function and increased cell death. Disease-associated proteins such as phosphorylated Tau, Aβ, LRRK2 G2019S, SOD1 G93A, and mutant Htt disturb the delicate mitochondrial dynamics, including fusion, fission, and trafficking, resulting in mitochondrial dysfunction. Manipulation of mitochondrial dynamics by genetic or chemical approaches may be a useful strategy to restore mitochondrial function and promote neuronal survival; (**B**) Mitochondrial dynamic abnormalities impair mitochondria transport and proper localization, leading to mitochondrial depletion in neurites and synapses and eventually neuronal death.

Considering the critical dependence of neuronal function and structure on mitochondrial function, it could be expected that, as highly polarized cells, neurons are particularly sensitive to alterations in mitochondrial dynamics (Figure 2A). For instance, neurons are vulnerable to the ablation of mitochondrial dynamic regulators such as Drp1, Mfn2, and Miro1 [56,66,67,68]. The deficiency of almost all mitochondrial fusion and fission regulators such as Drp1, OPA1, Mfn1, Mfn2, and Fis1 or the expression of dominant negative mutants of mitochondrial fusion and fission regulators such as Drp1 K38A and OPA1 K301A impairs mitochondrial movement and proper localization, leading to mitochondrial depletion in neurites and synapses and eventually to dendritic spine and synaptic loss [68,69,70] (Figure 2B). The crucial role of mitochondrial dynamics for neuronal function is echoed by the fact that genetic mutations in key regulators of mitochondrial dynamic cause dominantly inherited neurological diseases such as Mfn2 linked Charcot-Marie-Tooth type 2A (CMT2A), an inherited disorder characterized by the synaptic loss of motor neurons [71,72], and OPA1 linked optic neuropathy optic atrophy type 1 [73,74]. Below, we will review the current knowledge on mitochondrial dynamic abnormalities in various common neurodegenerative diseases.

### 3.1. Mitochondrial Dynamic Abnormalities in Alzheimer’s Disease (AD)

AD, first reported by Dr. Alois Alzheimer, is the most prevalent form of dementia in the elderly and is characterized by the progressive loss of neurons in brain regions critical for memory, learning, conscious thought, and language. The prominent pathological changes of AD patients include neurofibrillary tangles (NFTs), senile plaques (SPs), granulovacuolar degeneration, dystrophic neurites, Hirano bodies, and cerebrovascular amyloid [75]. As pathologic hallmarks, NFTs are intracellular aggregates composed of the hyperphosphorylated form of the microtubule-associated protein tau, while SPs are extracellular lesions made up of bundles of amyloid-β (Aβ) peptide fibrils [75]. Although many genetic, biochemical, and cellular studies have indicated a fundamental involvement of mitochondria in the development of AD [76], the likely contribution of mitochondrial dynamic abnormalities to mitochondrial and neuronal dysfunction has only come into focus over the last decade. Since the pioneering work by Hirai et al. [77], mitochondrial morphological changes manifested as fragmented mitochondria with damaged inner membrane structures have been increasingly reported in neurons in AD patients [78] and AD experimental models overexpressing or treated with Aβ or tau [78,79,80,81,82,83]. Consistently, these studies showed the altered expression of mitochondrial fusion and fission regulators such as Drp1, OPA1, Mfn1/2, and Fis1 or the changed Drp1 phosphorylation and S-nitrosylation post-translational modifications enhancing its GTPase activity. The majority of early-onset familial AD cases are associated with mutations in presenilins (PS) [84]. Similar to Aβ or tau, AD-associated PS mutants cause mitochondrial dysfunction in neuronal cells and transgenic mice [85,86,87,88]. An earlier study demonstrated the morphological change of mitochondria in cortical neurons from temporal and hippocampus of AD patient bearing PS1 E280A mutant [89], indicating the possible involvement of PS in regulating mitochondrial morphology. Of note, like a mitochondrial bioenergetic deficit, mitochondria fragmentation is an early feature preceding AD pathology in amyloid precursor protein (APP) transgenic animal models [82,90,91], suggesting the likely pivotal role of mitochondrial morphological abnormalities in disease progression. In further support of this notion, two most recent studies have reported that the inhibition of mitochondrial fragmentation by partial Drp1 deficiency is sufficient to alleviate mitochondrial dysfunction and synaptic loss in both APP and tau transgenic mouse models [92,93].

The coincident axonopathy and deficits in axonal transportation, as well as depletion of mitochondria or fusion and fission regulators in neurites in AD, suggest that mitochondrial and neuronal dysfunction in AD may also be attributed to the impairment of mitochondrial trafficking [78,94]. Mitochondria in neurons challenged with extracellular Aβ or neurons expressing APP show decreased motility and density in axons [78,95]. Similarly, tau, especially hyperphosphorylated and mutant (P301L) tau, disrupts mitochondrial transport in neuronal cells [96,97]. An Aβ or APP induced mitochondrial trafficking deficit could be alleviated by inhibiting mitochondrial fragmentation [78,83], indicating the impairment of mitochondrial movement possibly downstream of mitochondrial fragmentation. However, as mitochondrial morphology and trafficking and are highly interrelated [98], the direct involvement of mitochondrial trafficking in mitochondrial fragmentation and dysfunction in AD could not be ruled out, and future studies will still be interesting to test whether the restoration of mitochondrial transportation alone is able to prevent Aβ or tau induced mitochondrial and neuronal deficits.

The detailed mechanisms underlying mitochondrial dynamic abnormalities in AD have not yet been fully determined. The fact that both Aβ and tau can be present in mitochondria and interact with mitochondrial fusion and fission regulators such as Drp1 indicates that mitochondrial dynamic abnormalities may be direct rather than side effects of Aβ and tau induced neurotoxicity [80,81,99]. Alterations in ER and mitochondria association have been reported in AD patients and AD experimental models [100,101]. Parkin-mediated mitophagy has also been revealed as a potential mechanism responsible for mitochondrial trafficking deficits in AD patients and APP transgenic mice [102]. Given expanding evidence revealing the important role of autophagy in the pathogenesis of AD [103] and ER in regulating mitochondria dynamics [25,26], it appears that mitochondria dynamic abnormalities involve a myriad of factors and interaction between multiple signaling pathways. Nevertheless, defective mitochondrial morphology and transport in cell bodies, axons, and synaptic terminals in AD presumably cause local energy depletion, which in turn likely triggers or exaggerates neuron dysfunction and loss in AD. Therefore, abnormal mitochondrial dynamics may be an important pathway that contributes to mitochondrial dysfunction and neuronal dysfunction in the AD brain.

### 3.2. Mitochondrial Dynamic Abnormalities in Parkinson’s Disease (PD)

PD, first described by James Parkinson, is the second most common neurodegenerative disease, after Alzheimer’s disease, associated with characteristic motor impairments such as bradykinesia, resting tremor, and rigidity. Pathologically, PD is characterized by the progressive loss or degeneration of the dopaminergic (DA) neurons in the substantia nigra and the presence of intracytoplasmic inclusions (i.e., Lewy bodies) that are composed of a-synuclein in DA neurons [104]. As one breakthrough in PD research, MPTP (1-methyl-4-phenyl-1,2,3,6-tetrahydropyridine) was found to cause DA neurodegeneration and progressive and levodopa-responsive parkinsonism resembling sporadic PD [105]. MPP^+^ (1-methyl-4-phenylpyridium ion) is the active metabolite of MPTP concentrated within DA neurons via the dopamine transporter (DAT) specifically inhibiting OXPHOS complex I [106]. Interestingly, our previous study found that in a Drp1-dependent manner, MPP^+^ tipped the mitochondrial fusion and fission balance towards excessive fission to cause mitochondrial fragmentation concurrent with mitochondrial dysfunction but preceding neuronal death [107]; therefore not only indicating an unexpected important role of mitochondrial fusion and fission dynamics in mediating MPP^+^ toxicity but also directly linking mitochondrial dysfunction to mitochondrial dynamics in PD neurotoxin models.

Although the mitochondrial morphology in DA neurons of PD patients is still unclear, mitochondrial morphological changes accompanied by dysfunction have been consistently reported in peripheral cells from PD patients [108,109,110,111]. Over the last decade, mitochondrial fragmentation in experimental models inactivating proteins associated with recessive PD is one of the most highly studied topic in PD. Loss of PINK1, Parkin, or DJ-1 unanimously results in abnormal mitochondria morphology in muscle and DA neurons [112,113]. In line with these findings, mitochondrial fragmentation has been extensively reported in cell and animal models expressing proteins associated with autosomal dominant PD forms. For example, it was noted that mutant α-synuclein mislocalized to mitochondria to induce mitochondrial function and fragmentation [114,115]. In a Drp1-dependent manner, the expression of disease-causing leucine-rich repeat kinase 2 (LRRK2) mutants, the greatest known genetic contributors to PD so far, results in mitochondrial fragmentation [116]. Consistently, mitochondrial morphological alterations, as well as the changed expression of Drp1 and Fis1, were noted in LRRK2 G2019S knock in mice [117]. In addition, it has been reported that S-nitrosylation of Parkin regulates the expression of Drp1 to mediate mitochondrial fragmentation and neuronal loss in neurotoxin-based PD models [118]. In addition, our most recent study has shown that PD associated vacuolar protein sorting 35 (VPS35) mutants cause mitochondrial fragmentation by enhancing the clearance of inactive mitochondrial Drp1 complexes [31]. Of note, in most PD genetic models, the inhibition of mitochondrial fission alone is sufficient to greatly alleviate mitochondrial dysfunction, strongly suggesting the critical role of mitochondrial dynamics in mediating neurotoxicity induced by PD associated mutant proteins.

As reviewed above, mitochondrial fusion and fission dynamics are closely interrelated with trafficking. Not surprisingly, impaired mitochondria transport along axons was consistently noted in the PD toxin model and PD genetic models bearing PD-associated gene mutations [107,116]. Mitochondrial trafficking deficits in PD models could be prevented by the suppression of mitochondrial fragmentation [107,116], suggesting that mitochondrial movement impairment in PD could also be the downstream effect of altered fusion and fission dynamics. However, the identification of Parkin and a PINK1 mediated Miro1 degradation pathway strongly suggests a direct mechanism whereby mitochondrial trafficking could be regulated in PD [119].

Most of the identified proteins associated with PD, including α-synuclein, PINK1, Parkin, LRRK2, DJ-1, and VPS35, are localized to mitochondria or mitochondria-associated ER membranes (MAM) [31,116,120,121,122,123], indicating that these PD associated proteins might be regulators of mitochondrial dynamics, including fusion, fission, and trafficking in general. In support of this notion, Parkin and PINK1 mediate the degradation of several key dynamic regulators such as Drp1, Mfn1, Mfn2, and Miro1 [119,124,125,126,127]. Further, LRRK2 and VPS35 also physically interact with Drp1 to regulate mitochondrial dynamics and function [31,116]. Of note, missense mutations in mitochondrial fusion key regulator OPA1 (G488R, A495V) have been identified in patients with syndromic parkinsonism [128]. Not surprisingly, increasing evidence supports the idea that impaired mitochondrial dynamics are likely a common mechanism leading to mitochondrial and neuronal dysfunction and degeneration in PD [129].

### 3.3. Mitochondrial Dynamic Abnormalities in Amyotrophic Lateral Sclerosis (ALS)

ALS, also called Lou Gehrig’s disease, is the most common of the five motor neuron diseases characterized by progressive degeneration of motor neurons in the brain stem and spinal cord, resulting in primary symptoms, including muscle weakness and atrophy and difficulty in speaking, swallowing, and breathing. The prominent pathological hallmark of ALS is the presence of inclusion bodies in degenerating motor neurons, in which TDP-43 has been identified as the major component [130]. Abnormal mitochondrial morphology was noted in neurons and peripheral cells of sporadic or familial ALS patients [131,132,133], and, in the past decade, mitochondrial fragmentation has been well documented in ALS cell and animal models. Genetic mutations in Cu/Zn superoxide dismutase 1 (SOD1) were the first mutations identified for ALS. It has long been shown that mitochondria became fragmented concurrent with the changed expression of several mitochondrial fusion and fission regulators such as Drp1, OPA1, Mfn1, and Fis1 in experimental models expressing ALS-associated mutant SOD1, well preceding motor neuronal loss and symptom onset [134,135,136,137,138,139]. Similarly, neurons expressing ALS-associated mutant TDP-43 also show mitochondrial fragmentation and changed expression of mitochondrial fusion and fission regulators [138,140,141,142]. Consistent with these findings, mitochondrial fragmentation has recently been reported in neurons expressing ALS-associated mutant fused in sarcoma/translocated in liposarcoma (FUS), the other RNA/DNA binding protein associated with ALS [143]. Moreover, interestingly, like induced pluripotent stem cell (iPSC)-derived human neurons bearing TDP-43 mutations, iPSC-derived human motor neurons bearing disease causing C9orf72 hexanucleotide expansions also demonstrate mitochondrial fragmentation [132,144]. It may be expected that there will be more emerging new studies reporting mitochondrial morphological abnormalities in ALS and ALS models [145].

Mitochondria accumulate in the soma and proximal axon hillock of spinal cord motor neurons of sporadic ALS patients [146]. Consistent with this, similar abnormal mitochondrial clusters in proximal axons or around the peri-nuclear area were also noted in transgenic animals expressing ALS-associated SOD1 mutants and TDP-43 mutant [138,142,147,148,149,150], strongly suggesting impaired mitochondrial transportation in ALS. Along this line, cultured neurons expressing ALS-associated SOD1 or TDP-43 mutant indeed showed deficits in mitochondria axonal trafficking [138,142,151,152]. Notably, we have shown that the expression of Miro1 is significantly decreased in the spinal cords of ALS patients and mutant SOD1 and TDP-43 transgenic mice [153], therefore indicating the possibility that Miro1 downregulation likely contributes to mitochondrial movement abnormalities in ALS and ALS experimental models.

Wild type or mutant SOD1 can be located on or accumulate in the mitochondrial outer membrane, intermembrane space, and even matrix [154,155,156,157]. Our most recent study has revealed that wild type or mutant TDP-43 resides in the mitochondrial inner mitochondrial membrane facing matrix [132,142]. Likewise, RNA-binding protein FUS/TLS (Fused in Sarcoma/Translocated in Sarcoma, FUS) can enter mitochondria and interact with mitochondrial chaperonin HSP60 in the matrix [143]. Although without definitive evidences, the presence of ALS-associated SOD1, TDP-43, and FUS in mitochondria indicates the possibility of direct association of them with mitochondrial fusion, fission, and trafficking machineries. The inhibition of mitochondrial fission by overexpression of inactivated Drp1 or Mfn2 prevents mitochondrial trafficking deficits in motor neurons expressing disease causing mutant SOD1 or TDP-43 [135,142], implying that, in addition to Miro1 deficiency, the mitochondrial fusion and fission dynamic abnormalities may also be responsible for impaired mitochondrial movement in ALS. It is noteworthy that TDP-43 can be imported into mitochondria and directly interfere with OXPHOS complex assembly. Considering the close interplay between mitochondrial dynamics and bioenergetics [64,65], the likely indirect effects of ALS associated proteins on mitochondrial dynamics could be expected. Along this line, unlike other neurodegenerative diseases, the physical association between ALS associated proteins and mitochondrial dynamic regulators has not been reported. Therefore, mitochondrial fragmentation in ALS patients and experimental models is likely the downstream event or consequence of disease onset. However, we and other groups reported that the inhibition of mitochondrial dynamics abnormalities could improve mitochondrial and neuronal dysfunction caused by mutant SOD1 or TDP-43 in in vitro cultured neurons. Further studies will be tempting to investigate the role of mitochondrial dynamic abnormalities in the disease progression of ALS.

### 3.4. Mitochondrial Dynamic Abnormalities in Huntington’s Disease (HD)

HD is a dominantly inherited neurodegenerative disease involving progressive psychiatric, cognitive, and motor symptoms with a wide spectrum of other signs. HD is caused by CAG (cytosine-adenine-guanine) trinucleotide repeat expansion mutations, encoding a polyglutamine tract in the N-terminus of the Huntingtin (Htt) protein [158]. The prominent pathological features of HD include the extensive loss or degeneration of neurons, mainly in the striatum and cerebral cortex, and the presence of intracellular inclusion bodies made up of ubiquitinated or truncated Htt containing polyglutamine [159]. Mitochondrial fragmentation has been reported in peripheral cells of HD patients [49,160]. Consistently, significant changed expression or post-translational modifications of mitochondrial fusion and fission regulators such as Drp1, Mitofusions, and Fis1 was also observed in the brains of patients with HD [160,161,162]. For example, increased Drp1 S-nitrosylation were observed in the striatum of a transgenic mouse model of HD and HD patients, which correlated with excessive mitochondrial fragmentation followed by loss of dendritic spines, signifying synaptic damage [163]. Not surprisingly, a growing number of studies have shown that mitochondrial morphology becomes abnormal in experimental models for HD. For instance, mitochondrial fragmentation following mitochondrial dysfunction was observed in neuronal cells treated with 3-nitropropionic acid (3-NP), a mitochondrial complex II inhibitor causing progressive striatal neurodegeneration and locomotor deterioration resembling HD [164,165,166]. Further, iPSC-derived human GABAergic neurons from an HD patient or neuronal cells expressing Htt protein containing expanded polyglutamine tracts also displayed mitochondrial fragmentation [49,160]. Of note, in these HD toxin and genetic models, defective mitochondrial movement was also observed, suggesting the likely systematic impairment of mitochondrial dynamics in HD.

In 3-NP toxin models, mitochondrial dynamic abnormalities could be prevented by antioxidant treatment, indicating that mitochondrial dynamic changes might be the consequence of impaired mitochondrial biogenetics [164,165]. However, recent studies of the interaction between disease causing mutant Htt and mitochondrial dynamic regulators have revealed a likely direct interplay between Htt and mitochondrial dynamics. For example, the expression of S-nitrosylated Drp1 is increased in HD patients and HD transgenic mice expressing human mutant Htt [163,167]. Interestingly, mutant Htt can directly interact with Drp1 on mitochondria [49,160]. The inactivation of Drp1 or the blockage of Drp1 and Fis1 interaction alleviated mitochondrial dynamic abnormalities in neurons expressing mutant Htt, strongly suggesting the direct involvement of mutant Htt in mitochondrial fusion and fission dynamics. Mutant Htt has also been reported to selectively sequester and inactivate motor proteins such kinesis and dynactin or disrupt the association of motor proteins with microtubules by interacting with HAP1 to further result in impaired mitochondrial trafficking [168,169], thereby indicating a direct relationship between mutant Htt and mitochondrial trafficking. Taken together, mutant Htt has multiple effects on mitochondrial dynamics, and the impaired mitochondrial and neuronal function through mitochondria dynamics is likely a primary pathway contributing to the onset and progression of HD.

### 3.5. Perspective: Mitochondrial Dynamics as Common Therapeutic Targets for Neurodegeneration

Mitochondrial dynamics are very sensitive to physiologic stresses or pathologic stimuli, and mitochondrial dysfunction is a prominent early feature in a wide range of neurological disorders, including cerebral ischemia, stroke, brain trauma, and especially neurodegenerative diseases such as ALS, AD, PD, and HD [8,170,171] (Figure 2A). Not surprisingly, increasing evidence suggests that the impaired mitochondrial dynamics is an important pathological mechanism in these devastating diseases. Over the last decade, multiple studies have already demonstrated the feasibility of using the inhibition of mitochondrial fragmentation as a novel approach to prevent neuronal loss and even improve behaviors in different experimental models for neurodegenerative diseases. For example, two recent studies have shown the similar protective effects of mitochondrial fission inhibition via Drp1 deficiency on mitochondria and neurons in tau and APP transgenic animal models for AD [92,93]. Recombinant adeno-associated virus expressing the dominant negative Drp1 mutant or Mdivi-1, a small molecular inhibitor of Drp1, has been reported to inhibit mitochondrial fragmentation, restore dopamine release, and prevent DA neuron loss in PD animal models [172]. In addition, P110, an inhibitory peptide blocking Drp1 and Fis1 interaction, attenuates HD-associated neurotoxicity and behavior deficits [173] (Figure 2A).

## 4. Conclusions

AD, PD, ALS, HD, and many other neurodegenerative diseases are all characterized by the progressive degeneration of neurons in the central nervous system. Currently, there is no cure or effective treatment for these devastating diseases. Due to their complexity and intense need for mitochondria, neuronal function and survival highly depends on mitochondrial function. Along with our better understanding of the molecular basis of mitochondrial dynamics and their crucial role in regulating mitochondrial and neuronal function, mitochondrial dynamics have been increasingly recognized as a common and early feature likely responsible for mitochondrial and neuronal dysfunction in a wide range of neurodegenerative diseases [174]. Although the widespread presence of mitochondrial dynamic abnormalities and dysfunction in various neurodegenerative diseases suggest that the cause of altered mitochondrial dynamics could be multifactorial, emerging studies have implied that the manipulation of mitochondrial dynamics may be a common therapeutic approach to improve mitochondrial and neuronal function and prevent neurodegeneration.
